# An Effective Hybrid Routing Algorithm in WSN: Ant Colony Optimization in combination with Hop Count Minimization

**DOI:** 10.3390/s18041020

**Published:** 2018-03-29

**Authors:** Ailian Jiang, Lihong Zheng

**Affiliations:** 1 Department of Computer Science, Taiyuan University of Technology, Taiyuan 030600, China; ailianjiang@126.com; 2 School of Computing and Maths, Charles Sturt University, Wagga Wagga, NSW 2678, Australia

**Keywords:** wireless sensor network (WSN), routing algorithm, ant colony optimization (ACO), minimum hop count, network lifetime, network load balancing, optimal path, mutation strategy, dynamic energy threshold strategy

## Abstract

Low cost, high reliability and easy maintenance are key criteria in the design of routing protocols for wireless sensor networks (WSNs). This paper investigates the existing ant colony optimization (ACO)-based WSN routing algorithms and the minimum hop count WSN routing algorithms by reviewing their strengths and weaknesses. We also consider the critical factors of WSNs, such as energy constraint of sensor nodes, network load balancing and dynamic network topology. Then we propose a hybrid routing algorithm that integrates ACO and a minimum hop count scheme. The proposed algorithm is able to find the optimal routing path with minimal total energy consumption and balanced energy consumption on each node. The algorithm has unique superiority in terms of searching for the optimal path, balancing the network load and the network topology maintenance. The WSN model and the proposed algorithm have been implemented using C++. Extensive simulation experimental results have shown that our algorithm outperforms several other WSN routing algorithms on such aspects that include the rate of convergence, the success rate in searching for global optimal solution, and the network lifetime.

## 1. Introduction

Lifetime is a fundamental criterion of wireless sensor networks (WSNs). Most existing methods for prolonging the lifetime of WSNs focus on the issues of device placement, data processing, routing, topology management, and device control. Among them, routing plays a significant role in optimizing energy efficiency leading to a longer network lifetime [1,2,3]. However, WSN routing protocols often face substantial challenges. These challenges come from the stringent energy constraint of each sensor node, from the inherent unreliability of wireless communication, from the dynamic nature of the network, and from the possibly large scale of the network. Thus, low cost, high reliability and easiness to maintain are always the key criteria for the design of a WSN routing protocol. The concept of searching for the shortest path between the source and destination nodes is widely used in WSN routing algorithm design. The source node transmits the data to the base station, also referred as sink node through a range of relay nodes, and short transmission distance between nodes corresponds to a low dissipation of energy. Therefore, the selection of the shortest path for data transmission is critical in reducing the energy consumption of the nodes and improving the stability of the WSN. Among various different approaches, ant colony optimization (ACO)-based WSN routing algorithm and minimum hop count (MHC)-based WSN routing algorithm [4,5] show superior capability in finding the shortest path. The paper conducts a thorough analysis of the advantages and disadvantages of the existing ant colony optimization WSN routing algorithms, as well as minimum hop count-based WSN routing algorithms. Then we integrate the two algorithms as a hybrid WSN routing algorithm, comprehensively taking into consideration several critical factors specific to WSN, including energy constraint of sensor nodes, network load balancing, and dynamic network topology. Hereinafter the proposed algorithm refers to ant colony optimization in combination with hop count minimization WSN routing algorithm (ACOHCM).

The new hybrid algorithm, ACOHCM, has unique superiority in terms of topology maintenance, searching the optimal path, and the network load balancing. A common topology of WSNs has a group of nodes that includes a sink node and a number of sensor nodes. ACOHCM borrows the idea of hop count-classification in MHC algorithms. The sensor nodes are firstly marked as different gradations according to their hop count distance to the sink node. So a node will prefer the neighbor nodes who have a lower hop count than the node itself, that is, which are closer to the sink node, as the alternative next hop nodes for data transmission. Therefore, the new proposed algorithm reduces attempts at choosing the nodes which are farther from the sink node to transfer the data. So, the optimal path can be identified much more quickly, and the convergence speed of the algorithm increases rapidly, compared with the conventional ant colony optimization WSN routing algorithms. In the aspect of searching for the optimal path, which is one of the challenging issues in a dynamically changing environment of WSN, we integrate the two algorithms, taking advantages of the two in finding the shortest path and overcoming their disadvantages which will be reviewed in the part of related works. The paper defines a WSN-specific heuristic factor for path selection and proposes a new pheromone update formula. The mutation strategy is also introduced to the algorithm to improve the success rate of searching for the global optimal solution for the optimal path. Regarding balancing the network load, many of the existing routing algorithms use a multi-path routing approach to achieve load balancing. The issue is the fact that using the minimum energy consumption path all the time depletes the energy of nodes on that path. Hence, it encourages the use of multiple paths with a certain probability, which favors battery depletion of different nodes at a comparable rate. However, this kind of multi-path routing approach must take into account the resulting communication interference problem. Different from the multi-path approach, we propose a dynamic energy threshold strategy. This strategy strictly takes control of the energy consumption of each sensor node in an effort to achieve a similar rate of energy depletion of each node so as to avoid premature death of some nodes due to energy depletion. ACOMHC also uses this strategy to trigger route update to prevent the sensor nodes on the routing path from over-consuming of energy and premature death due to data forwarding so as to resolve the problem of “Hot Spot”. By equalizing the data transmission workload, ACOMHC achieves load balancing. Simulation experiment results verify that ACOHCM algorithm has high performances in these respects, including the convergence speed, the success rate of searching for the optimal solution and the network lifetime.

The structure of this paper is as follows. In Section 2 we give a review on the ACO-based algorithms and MHC-based algorithms. Section 3 presents our system model and we discuss our proposed method in Section 4. Extensive simulation results and analysis are given in Section 5. Finally, we conclude in Section 6.

## 2. Related Works

Motivated by the foraging behavior of ants, bees and fishes, swarm intelligence (SI) has been developed and applied successfully to solve many optimization problems in real time as per Saleem and Di Caro [6]; Kuila and Jana [7]. Ant colony optimization (ACO), Particle Swarm Optimization (PSO) and Genetic Algorithm (GA) are three commonly used optimization algorithms in the family of SI. This section focuses on reviewing the strengths and weaknesses of WSN routing algorithms based on ACO, as well as the WSN routing algorithms based on minimum hop count.

### 2.1. Ant Colony Optimization Routing Algorithm

The Ant colony optimization (ACO) algorithm was proposed by Italian scholars Dorigo et al. [8], which imitates the behavior of ant colonies as ants search for the shortest path from their nest to the food source. The ants deposit a certain amount of pheromone on the path they traverse during the foraging process, and the posterior ants choose their path according to the pheromone intensity. Thus, the collective behavior of ant colony composed of a large number of ants shows a positive feedback of information:The shorter the path, the more ants will traverse it, producing the higher pheromone intensity, which will increase the probability of latter ants choosing the same path. Ants use this kind of information exchange among individual ants to find the shortest path for food. Classic ACO has been successfully applied in solving a number of scientific and industrial combinatorial optimization problems, such as small traveling salesman problem, vehicle routing problem, Quality of service (QoS) network routing, etc. Dorigo and Gambardella [9]; St$\tilde{u}$tzle and Hoos [10]; Jose et al. [11]; Zhang [12]; Zhan [13]. ACO routing algorithms work in a highly distributed way, and have properties such as adaptivity, robustness, and scalability. These characteristics make them particularly applicable to deal with aforementioned challenges in WSN routing.

Over the last decade, a significant number of routing algorithms based on ACO have been developed to address the challenges in WSN. Examples include the approaches from various research papers [2,14,15,16,17,18,19,20,21,22,23]. Because limited energy is the most critical issue affecting WSNs performance, many of these research works focus mainly on how to use the limited energy of WSNs to maximize the network lifetime, which has been seen as the all-important problem of WSN routing design. Yang et al. [18] proposed a novel multipath routing protocol (MRP) based on dynamic clustering and ACO. An improved ACO algorithm has been applied in searching for the optimal and suboptimal paths between the cluster head and the sink node. Furthermore, a load balancing function is included for dynamically selecting one path to transmit data with a probability that depends on path metrics, such as energy consumption in the path, residual energy, path length. Since the path is dynamically chosen, load balancing among the paths is guaranteed. MRP uses multiple paths to avoid a frequently used path on which thenodes with minimum energy consumption are located and keeps better balance of energy consumption among nodes, and then, prolongs the network lifetime. Khoshkangini et al. [19] combined ACO and Breadth First Search (BFS) to find the best and shortest path in order to improve data transmission with the minimum energy consumption, as well as reduce the probability of data loss. By applying BFS, the accurate selection of hops in the data transmission has been enhanced with minimum transmission time and the least energy consumption. Although it increases the search accuracy in finding the shortest path, implementation of BFS in the ACO introduces additional node memory and time consumption which are the weaknesses of the method. Kim et al. [20] Proposed an Inter-cluster Ant Colony Optimization algorithm (IC-ACO) that relies upon the ACO algorithm to identify the optimal path between a cluster head and the base station and effort has been made to minimize the attempts wasted in transferring the redundant data sent by the sensors which lie in the close proximity of each other in a densely deployed network. The paper provides us reference for routing of data packets in the densely deployed network. Sobral et al. [21] used a fuzzy system to estimate the route quality based on the number of hops and the energy level of the nodes that compose a route. The Ant Colony Optimization (ACO) algorithm is used to optimize the fuzzy rules in order to enhance the estimation quality of the routes, hence the energy efficiency of the network increases. The evaluation metric values, including the number of messages delivered to the sink node, an average cost of message, a packet loss rate, and the time of death of the first sensor node, have demonstrated the proposal is effective. Li et al. [22] utilized the ACO and borrowed an neural network (NN) rather than the greedy algorithm to build their chain of PEGASIS. The chain makes a path to send data to the BS more evenly distributed and reduces the total transmission distance much less. Therefore, it realizes energy consumption balancing between nodes. Liu [23] put forward an optimal-distance-based transmission strategy for lifetime maximization on the basis of the ACO, in which a local optimal transmission-distance acquirement mechanism is designed for both high energy efficiency and well energy balancing. Furthermore, a global optimal transmission-distance acquirement mechanism is provided to achieve energy depletion minimization for nodes with maximal energy consumption throughout the network. Mohajerani et al. [2] proposed an ACO-based routing algorithm called life time aware routing algorithm for wireless sensor networks (LTAWSN). It specifies its probability formula for reducing energy consumption of network nodes and also obtains more balanced transmission among the nodes and prolong the network lifetime. In addition, a new pheromone update operator is designed to integrate energy consumption and hop count together into routing choice.

From the above representative research works that have been reviewed, it is clear to see that, significant efforts have been made to exploit and modify the ACO to achieve the efficient use of energy or balanced use of energy. Eventually all these are necessary to maximize the network lifetime. However, when applying the ACO to WSN routing optimization, still there are the following shortages that decrease its performance and preclude its practical application.

(1) The searching time of these algorithms is long and the convergence speed of the algorithms is slow.
(2) The algorithm is prone to be trapped to a local optimum.
(3) When the global optimal path is found, the data will be transmitted through the optimal path all the way, causing the sensor nodes on the optimal path to deplete their energy at a faster pace and lead to the death of these nodes, and this phenomenon is called “Hot Spot”, which is very unfavorable to the life time of the network.

Sun and Tian [24] proposed a new hybrid method for route optimization in WSNs. For the convenience of reference, herein named ACO-GA. This method is based on a combination of modified ACO algorithm and genetic algorithm (GA). The initial solution is generated by the ACO as the population for the GA and next, the best solution is searched by further iterations of GA using selection, crossover and mutation. Thus it can accelerate the convergence speed of the ant colony algorithm, improve the efficiency and avoid the local optimum and precocity. Moreover, authors added a multi-path route for transmitting data. Simulation results showed that the new method was effective and has shown a better performance than ACO or GA individually. However, the computation complexity of such an algorithm increased, and the problem of energy equilibrium consumption was ignored. Ismkhan [25] pointed out three main drawbacks of the recent ACO algorithms. These include the implementation of pheromone is inefficient, the selection of next move by transition rules is very time consuming, and the local search used in the ACO operates without effective use of pheromone values. These drawbacks increased the computation time and space complexities of ACO, decreased its performance, and prevented it to be applied to large-scale instances. Then this paper suggested three new strategies to address those drawbacks.

In short, there are many important factors that influence the network performance dramatically to be considered when designing an effective routing algorithm for WSN, such as minimizing total energy consumption, balancing the network load, speeding up the convergence and the dynamic networking capability. The best practice is expected to be able to comprehensively take into account all these factors at the same time in order to address the challenges mentioned above in real-time applications.

### 2.2. Minimum Hop Count WSN Routing Algorithm

On the basis of the Directed Diffusion algorithm (DD) and the flooding algorithm, Han et al. [4] introduced the concept of hop count and proposed the minimum hop count (MHC) WSN routing algorithm. In the phase of data transmission, the algorithm determines the transmission path according to the hop count from the source node to the sink node. MHC routing algorithm has the potential advantages of shortest path, lowest energy consumption, and minimum time delay. However, the following two concerns disadvantages the original minimum hop count routing algorithm.

(1) The sink node periodically initiates flooding for route update. This kind of periodic flooding throughout the whole network may cause excessive cost and overhead in terms of energy due to redundant data transmissions.
(2) There is no reasonable strategy to choose the next hop nodes for data transfer in the process of data transmission but forwarding the same data through all the parent nodes, which generates a large quantity of redundant information in the network and accelerates the energy consumption of the network.

Chiang et al. [5] established routing tables for sensor nodes using a technique similar to the classical flooding method. In each node’s routing table, it includes its neighbor nodes. They are marked as parent nodes, sibling nodes or child nodes respectively by recording their hop count values, together with their ID numbers, energy levels and time slots. A node’s parent nodes are whose hop counts are one less than this node itself, sibling nodes are who have the same hop counts with this node itself, and the child nodes are whose hop counts are one more than this node itself. Based on the routing table, in accordance with the priority of the parent nodes and the sibling nodes, each sensor node can determine the best next-hop node which has the highest energy level. The proposed data routing protocol reduces some redundant data transmission by providing a next hop node selection approach.

Ho et al. [26] proposed an ACO-based ladder diffusion algorithm to solve the energy consumption and transmission routing problems in WSNs. The algorithm consists of two phases, namely the ladder diffusion phase and route-choosing phase. The ladder diffusion creates a ladder table for each sensor node, and the route-choosing phase integrates ACO to select and construct the route from a sensor node to the sink node. The proposed algorithm greatly reduces the energy consumption compared to the previous work by properly assigning the transmission route using the ACO. However, the algorithm has a main flaw that it does not consider the sensor nodes’ residual energy when choosing the relay node. So the algorithm is easy to face in the "Hot Spot" and "Energy Pole" problems.

Du et al. [27] modified Ho’s method as an energy aware ladder diffusion (EALD) routing algorithm. The EALD adopts the ladder diffusion process similar to [26] in the first step. In the second step of its transmitting phase, in order to solve the ’Hot Spot’ and ’Energy Pole’ problems, an energy aware strategy is used to dynamically adjust the routes of transmitting according to the nodes’ residual energy. The key strengths of this modified algorithm include low routing overhead, network load balancing and the extended network lifetime.

## 3. System Model

### 3.1. WSN Network Model

WSN is a self-organizing network that consists of a number of distributed wireless sensor nodes and a sink node. Sensor nodes can sense the environment, perform basic computations on the collected data, and communicate with neighboring nodes via a radio transmitter. The data collected by sensor nodes from a target area is finally forwarded to the sink node one hop by one hop for further processing. In our ACOHCM algorithm, there are 6 assumptions.

(1) All nodes in the network are randomly deployed in the monitoring area. After the deployment, the location no longer changes. The sink node has knowledge of the topology of the whole network.
(2) All sensor nodes in the network are homogeneous, i.e., the node’s computing power, communication capability, and initial energy are the same.
(3) All sensor nodes are energy-constrained and cannot be replenished, while the sink node can be supplemented.
(4) The radio transmission power of the node is adjustable, and the transmission power can be adjusted according to the distance.
(5) Like other related works, Packet error and overtime retransmission caused by link errors are not considered.
(6) The sink node periodically carries out data collection, and all sensor nodes forward the data according to their own routing.

### 3.2. Energy Consumption Model

In WSNs, a node’s energy consumed for data communication is much higher than other energy consumption, such as data sensing and data processing. That is to say, the major portion of a node’s energy is consumed for data communication including data transmission and data receiving. So, our research mainly focuses on the energy dissipation for data communication. We use the model discussed in Heinzelman and Chandrakasan [28] for the radio hardware energy dissipation, where the transmitter dissipates energy to run the radio electronics and the power amplifier, and the receiver dissipates energy to run the radio electronics. Thus, to transmit a k-bit data packet to the receiver at distance *d*, it takes the energy cost as shown in the following Formula (1):

(1) $$E_{T\gamma }\left(k,d\right)=E_{T\gamma -elec}\left(i,j\right)+E_{T\gamma -amp}\left(k,d\right)=\left\{\begin{array}{cc} kE_{elec}+k\epsilon_{fs}d^{2}, & d\;<\;d_{0}\\ kE_{elec}+k\epsilon_{mp}d^{4}, & d\;\geq \;d_{0} \end{array}\right.$$

The energy consumption for data transmission consists of two parts: the electronics energy consumption, denoted as $E_{T\gamma -elec}$ and the power amplifier energy consumption, denoted as $E_{T\gamma -amp}$. The power amplifier energy consumption is related to transmission distance and the environment, divided into the free space model ($d^{2}$ power loss) and multipath fading ($d^{4}$ power loss) channel models, depending on the distance between the transmitter and receiver. Power control can be used to invert this loss by appropriately setting the power amplifier—if the distance is less than a threshold $d_{0}$, the free space model is used; otherwise, the multipath model is used.

On another hand, to receive these data, the cost is:

(2) $$E_{Re}\left(k\right)=E_{Re-elec}\left(k\right)=kE_{elec}$$

The Formula (2) contains only the electronics energy consumption. $E_{elec}$ is the energy dissipation of per bit for transmitter or receiver. Based on Formulas (1) and (2), energy consumed for data transmission is higher than that of data reception. The energy consumption for data transmission relies mainly on the distance squared whereby the farther between the sender and the receiver, the higher the energy is consumed to transmit the data.

## 4. ACOHCM Routing Algorithm

The goal of the ACOHCM routing algorithm is to maximize the lifetime of the WSNs. Moreover, an effective routing algorithm must converge quickly and have high rate of successful searching for the best solution for the optimal path, as well as low overhead in route discovery, route update and maintenance. Therefore, the ACOHCM algorithm uses the hop count-classification network topology, which is a kind of planar network topology. It is based on the node location information, so it is easy to maintain. The routing protocol on this topology has the advantages of simple structure and low maintenance overhead. In order to search for an optimal path with minimum total energy consumption and balanced energy consumption of each node, a dynamic energy threshold strategy and a mutation strategy have been proposed. Especially, we revise the heuristic factor and replace a new pheromone update strategy in the probabilistic node state transition formula for path selection. The new algorithm takes into comprehensive account energy efficiency, load balancing, convergence speed, and routing reliability as well as low maintenance. Energy efficiency here refers to the ratio of total packet delivered at the sink node to the total energy consumed by the network’s sensor nodes on the data transmission path during the data transmitting process (Kbit/J). So it is expected to minimize the total energy consumption of the sensor nodes on the routing path. In addition, It is expected to reduce the redundant transmissions involved in route discovery and data delivery to reduce the energy consumption of a node.

### 4.1. Hop Count-Classification Network Topology and Maintenance

ACOMHC algorithm is developed considering both the ant colony optimization algorithm and the minimum hop count WSN routing algorithm. Before the ants start off, the algorithm examines the location information of each node in the network and uses the idea of hop count-classification in MHC algorithm as a reference to mark all the nodes by their hop count values. The definition for the hop count of a node is as follows.

**Definition** **1.** *The hop count of the sink node is 0, the hop count of the other nodes is the smallest hop count among their neighbor nodes plus 1*.

**Figure 1:**
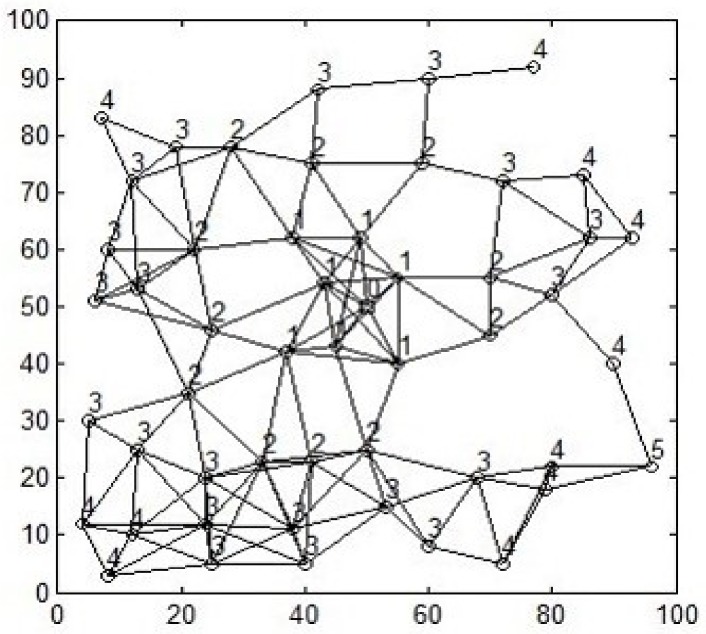
Hop count-classification network topology in an area of 100 m × 100 m.

After the hop count of each node is marked, the nodes in the whole network form a hop count-classification network topology as shown in Figure 1. The square at the center of the figure represents the sink node and the rest are ordinary sensor nodes. The number on the node indicates the hop count of that node. According to the definition of the hop count, any *h*-hop (*h* ≥ 2) nodes cannot transmit data to the nodes whose hop counts are *h* ± 2 without starting power amplifier. So the best next hop nodes of a *h*-hop (*h* ≥ 1) node must be h−1 hop nodes without considering the energy of the nodes. Therefore, the hop count-classification topology can greatly reduce the number of alternative nodes when choosing the next hop node, which accelerates its convergence speed and reduce the time complexity of the algorithm.

In addition, topology maintenance is responsible for the maintenance of the hop count-classification network topology. The process of topology maintenance will start when any network topology changes in the case of a sensor node failure or wireless link breakage. Any topology change will affect the nodes’ hop count values dynamically. Hence, hop count values need to be updated timely. Correspondingly, the route also needs to be updated.

### 4.2. Dynamic Energy Threshold Strategy

In order to balance the energy consumption of the entire network, this paper proposes a dynamic energy threshold strategy. The basic idea can be stated as follows. Assumed that the initial energy value of each node is *C*. The energy threshold θ and the lower bound energy threshold $\theta_{min}$ are set for all nodes during the initialization. They are both less than *C*, and $\theta_{min}\leq \theta$. When an ant searches for the next hop node, $\theta_{c}$, a copy of the energy threshold θ, is created, then the algorithm seeks the nodes whose current energy are greater than $\theta_{c}$ as the alternative next hop nodes according to the priority order of parent, sibling, child nodes in the set of neighbor nodes of the node *i* where the ant is located. Those nodes whose current energy are less than or equal to $\theta_{c}$ are only responsible for sensing data and sending data collected by themselves. They do not transfer the data from the other nodes. If the current energy of every neighbor node of the node *i* is less than or equal to $\theta_{c}$, $\theta_{c}$ will be updated dynamically according to the Formula (3) as follows.

(3) $$\theta_{c}=\left\{\begin{array}{cc} \lambda \times \theta_{c}, & \lambda \times \theta_{c}\;>\;\theta_{min}\;\\ \theta_{min}, & \lambda \times \theta_{c}\;\leq \;\theta_{min}\; \end{array}\right.$$

λ represents the extent of decrease of the threshold, and 0<λ<1. If the current energy of every neighbor node of the node *i* reaches the lower bound energy threshold $\theta_{min}$, the power amplifier is started and the alternative next hop nodes are selected from the nodes with h±2 hops, here *h* is the hop count of the node *i*.

During the running of the network, the energy of the nodes is constantly consumed. If the remaining energy of 85 percent of the nodes in the network have reached the energy threshold θ. θ is updated dynamically according to the Formula (4) as follows. Then $Set_{reach\_th}$ is emptied. Here $Set_{reach\_th}$ is a set used to store the nodes whose remaining energy have reached or lower than the energy threshold.

(4) $$\theta =\left\{\begin{array}{cc} \lambda \times \theta , & \lambda \times \theta \;>\;\theta_{min}\;\\ \theta_{min}, & \lambda \times \theta \;\leq \;\theta_{min}\; \end{array}\right.$$

When the remaining energy of a node *i* reaches the energy threshold of θ and $i\notin Set_{reach\_th}$, the route update is triggered. That is, the sink node will initiate a new route discovery process, and the node *i* joins into $Set_{reach\_th}$.

### 4.3. Probabilistic Selection for the Best Next Hop Node

The probabilistic node state transition formula in the ant colony optimization algorithm mainly consists of two key components: pheromone intensity on alternative paths and the heuristic factor. ACO utilizes those two parameters: search experiences represented by pheromone intensity, and domain knowledge in form of heuristic factor information to accelerate the search process. Pheromone intensity values are the results of the long-term collective learning of good paths from ants’ actions, while the heuristic values reflect the node’s local situation. The heuristic factor changes in the different fields of application. For the wireless sensor network routing optimization, we comprehensively take into consideration the residual energy of the alternative next hop nodes and the distance between node *i* and *j*, and using the weighting factors $\xi_{1}$ and $\xi_{2}$ ($\xi_{1}+\xi_{2}=1$ ) to indicate their role importance respectively, therefore the heuristic factor $\eta_{ij}$ in ACOHCM algorithm is defined as Formula (5).

(5) $$\eta_{ij}=\frac{E_{j}}{C}\xi_{1}+\frac{d_{sum}}{d_{ij}}\xi_{2}$$

From (5) it is clear to see that $\eta_{ij}$ is intended to favor the selection of a node with higher level of residual energy and with shorter communication distance between the sender and receiver so as to save the data transmission energy consumption and balance the energy usage. Where *C* is the initial energy value of the sensor nodes; $E_{j}$ is the remaining energy value of the node *j*; $d_{ij}$ is the distance between node *i* and node *j*; $d_{sum}$ is the sum of the distances between all alternative next hop nodes and the source node. Thus, the probabilistic node state transition formula indicating the probability of the ant *k* transferring from node *i* to node *j* at time *t* is shown as Formula (6).

(6) $$P_{ij}^{k}\left(t\right)=\left\{\begin{array}{cc} \frac{\tau_{ij}^{\alpha }{\left(\frac{E_{j}}{C}\xi_{1}+\frac{d_{sum}}{d_{ij}}\xi_{2}\right)}^{\beta }}{\sum_{\mu \in allowed_{k}}\tau_{i\mu }^{\alpha }{\left(\frac{E_{\mu }}{C}\xi_{1}+\frac{d_{sum}}{d_{i\mu }}\xi_{2}\right)}^{\beta }} & j\;\in \;allowed_{k}\\ 0 & \mathrm{otherwise} \end{array}\right.$$

where $\tau_{ij}$ is the pheromone value on the path $L_{ij}$; α and β indicate the relative influence of cumulative pheromone on the path and the local heuristic factor respectively on path selection for an ant; $allowed_{k}$ is the set of next hop nodes allowed to be selected for ant *k*. According to Formulas (5) and (6), the more residual energy the alternative node *j* has, and the shorter the distance from the node *j* to the node *i* is, the larger the value of the heuristic factor will be, and the bigger the probability of the node *j* will be selected.

### 4.4. Pheromone Update Strategy

The pheromone update strategy in the ACOMHC algorithm is as follows: In the end of each iteration, only the pheromone values of the optimal path in this iteration are updated, and the pheromone values of the other paths are partially volatilized; In order to avoid the unlimited growth or reduction of the pheromone on the certain paths, ACOHCM limits the pheromone values by setting the upper pheromone limit $\tau_{max}$ and the lower pheromone limit $\tau_{min}$. If the updated pheromone exceeds the upper or lower limit, the pheromone is assigned upper pheromone limit or lower pheromone limit. Therefore, the ants have probability to try taking other paths, which increases the ability of the algorithm to find the global optimal solution and prevents the algorithm from prematurely converging to the local optimal solution. Using this pheromone update strategy, obviously can speed up the convergence of the algorithm, as well as overcome the stagnation of ACO algorithm.

The improved pheromone update formula is defined as in Formula (7).

(7) $$\tau_{ij}\left(t+n\right)=\left\{\begin{array}{cc} \left(1-\rho \right)\tau_{ij}\left(t\right), & L_{ij}\;\mathrm{is}\;\mathrm{not}\;\mathrm{on}\;\mathrm{the}\;\mathrm{optimal}\;\mathrm{path}\;\&\;\left(1-\rho \right)\tau_{ij}\left(t\right)>\tau_{min}\;\\ \left(1-\rho \right)\tau_{ij}\left(t\right)+\Delta \tau_{ij}, & L_{ij}\;\mathrm{is}\;\mathrm{on}\;\mathrm{the}\;\mathrm{optimal}\;\mathrm{path}\;\&\;\tau_{min}\;<\;\left(1-\rho \right)\tau_{ij}\left(t\right)\;<\;\tau_{max}\\ \tau_{max}, & L_{ij}\;\mathrm{is}\;\mathrm{on}\;\mathrm{the}\;\mathrm{optimal}\;\mathrm{path}\;\&\;\left(1-\rho \right)\tau_{ij}\left(t\right)+\Delta \tau_{ij}\;\geq \;\tau_{max}\\ \tau_{min}, & L_{ij}\;\mathrm{is}\;\mathrm{on}\;\mathrm{the}\;\mathrm{optimal}\;\mathrm{path}\;\&\;\left(1-\rho \right)\tau_{ij}\left(t\right)+\Delta \tau_{ij}\;\leq \;\tau_{min},\mathrm{or}\\ & L_{ij}\;\mathrm{is}\;\mathrm{not}\;\mathrm{on}\;\mathrm{the}\;\mathrm{optimal}\;\mathrm{path}\;\&\;\left(1-\rho \right)\tau_{ij}\left(t\right)\;\leq \;\tau_{min} \end{array}\right.$$

$\Delta \tau_{ij}=\sum_{k=1}^{m}\Delta \tau_{ij}^{k}$, *m* is the number of the ants:

(8) $$\Delta \tau_{ij}^{k}=\left\{\begin{array}{cc} \frac{Q}{L_{k}}, & \mathrm{ant}\;k\;\mathrm{goes}\;\mathrm{pass}\;\mathrm{the}\;\mathrm{path}\;L_{ij}\\ 0, & \mathrm{others} \end{array}\right.$$

ρ(0<ρ<1) is a pheromone evaporation factor, and (1−ρ) is pheromone residual factor; $\Delta \tau_{ij}$ is the pheromone that the ants deposit on the path $L_{ij}$ in the current iteration; $\Delta \tau_{ij}^{k}$ is the pheromone that the ant *k* deposits on the path $L_{ij}$ in the iteration; *Q* is a constant and it affects the convergence speed of algorithm to a certain extent; $L_{k}$ is the length of path that the ant *k* traverses in the iteration. It is thus clear that the smaller the $L_{k}$ is, the more pheromone that the ant deposits on the path, which is helpful in searching for the optimal path.

### 4.5. Mutation Strategy

In order to improve the global optimization ability of the algorithm, and prevent the algorithm from stopping at an arbitrary local optimum, ACOHCM algorithm uses the mutation strategy to control the ants path finding. Each ant is assigned a mutation probability, assuming δ=10% at initialization. Generally, when an ant chooses the next hop node, it firstly looks up its neighbor table and puts those eligible nodes into $Set_{cand}$, a set of alternative next hop nodes. Then it determines which is the next hop node according to the probabilistic node state transition Formula (6). However, when the mutation occurs, the ants will choose a node from $Set_{cand}$ according to the roulette rule as the next hop node.

During the iteration of searching for the optimal path, if the optimal solution in the current iteration is no better than the previous one, the algorithm will increase the mutation probability to δ=40%. So the ant has a larger probability to try other paths to improve the searching ability of the algorithm. The mutation probability of the ant is not re-adjusted to the original value of δ=10% until the algorithm finds a better solution than the solution in the previous iteration.

### 4.6. Implementation of ACOHCM Algorithm

The implementation procedure of the ACOHCM algorithm includes three main steps, namely initialization, network topology generation and routing finding. The pseudo code is given in Algorithm 1.

**Algorithm 1** ACOHCM algorithm.**function**    1.Initialization    (Set initial energy value, energy threshold, lower energy threshold, communication radius of the node, lower pheromone limit, upper pheromone limit, mutation probability of the ant)    2. Network topology generation    According to the nodes’ location information, the hop count-classification network topology is generated    3. Route finding    **for** node *i* = 1 to Number_of_Nodes) **do**        **for** iter = 1 to Numbre_of_Iteration **do**           **for** ant = 1 to Numbre_of_Ants **do**               {               3.1 Get the set of the alternative next hop nodes according to the dynamic energy threshold strategy.               3.2 Determine the best next hop node based on the improved probabilistic node state transition Formula (6) and the mutation strategy.               3.3 Update Location_of_ant.               } While (the ant has not reached the sink node)               3.4 Calculate the total energy consumption of data transmitting along the path by which the ant goes.           **end for**           3.5 Choose the path with minimal total energy consumption among the paths that *m* ants find out.           3.6 Ants feedback backwards. Update the pheromone values according to Formula (7).        **end for**        3.7 Output the optimal path from the node i to the sink (whose total energy consumption is minimal, the number of hops is least, and the energy consumption is balanced on the path)    **end for**    3.8 Output the routing information of every node in the network.**end function**

The choices of the parameters α,β,ρ in Formulas (6) and (7) have important effects on the ant routing process [29]. Generally speaking, the greater α is, the bigger the probability that an ant will traverse the path that has been passed through in the past, which will lead the ant colony algorithm to fall into the local optimal solution earlier. The smaller α is, the smaller the effect of the pheromone with the higher the randomness of the path selection, which will increase the searching time, slowing the convergence speed of the algorithm. The greater β is, the bigger the probability that an ant chooses the node with high residual energy and shorter distance. However, if β is too large, it will suppress the effect of the pheromone; The smaller β is, the higher the randomness of the next hop node selection. Pheromone evaporation factor ρ is another crucial parameter. The greater ρ is, the stronger the role of the positive feedback is, which will increase the likelihood of the traversed path being re-selected, while decrease the randomness of searching. On the contrary, the smaller ρ is, the bigger the randomness of the search becomes, and the stronger the global search capability of the algorithm becomes.

When the wireless sensor network is started, the ACOMHC algorithm is run to generate the routing information for each node, and all nodes perform data forwarding according to the routing information.

### 4.7. Analysis of the Convergence Speed of the Algorithm

The ant colony optimization algorithm, as a swarm intelligence randomized search heuristics (RSHs), is characterized by randomness, teamwork, universality and so on. With such features the ant colony optimization algorithms show a good way to simulate complex dynamic behavior. However, the complex stochastic process caused by such a complex dynamic behavior makes ant colony optimization algorithms experience difficulty in their theoretical analysis, such as convergence analysis and time complexity analysis [30]. Gutjahr [31] developed a Graph–based Ant System, a metaheuristic which can treat arbitrary static combinatorial optimization problems, and for the first time proved that under certain conditions (by a suitable choice of the parameters of the heuristic), the solutions generated in each iteration of this Graph–based Ant System converge with a probability that can be made arbitrarily close to one to the optimal solution of the given problem instance. Stutzle and Dorigo [32] presented a short convergence proof for an ant colony system (ACS) and max-min ant system (MMAS). The convergence analysis theory only tells us that ant colony optimization algorithms have the possibility of finding the global optimal solution, and it is hard to evaluate the performance of actual algorithms as what we expected. Hence, it is necessary to analyse the convergence speed of ant colony algorithms so that we can know how quick the algorithms are to find the optimal solution. Dedigo in 2005 explicitly listed the research on convergence speed for ACO algorithms as the first open problem of ACO research field [33]. Moreover, Neumann [34] conducted research about the influence of the pheromone evaporation factor ρ on the time complexity of ACO algorithm. He showed that for $\rho =O(n^{-1-\epsilon })$, ϵ>0, the optimization time of 1-ANT on OneMax is $2^{\Omega (n^{\epsilon /3})}$ with probability of 1–$2^{-\Omega (n^{\epsilon /3})}$, whereas for $\rho =O(n^{-1+\epsilon })$, ϵ>0. An $O(n^{2})$ upper optimization bound holds with overwhelming probability of 1–$2^{-\Omega (n^{\epsilon /3})}$. This reveals a phase transition phenomenon from exponential expected optimization time to polynomial expected optimization time of 1-ANT on OneMax occurring for a fast decrement of ρ. Doerr et al. [35] also analyzed rigorously the influence of ρ has on the performance of 1-ANT algorithm. Lower bounds of optimization time for 1-ANT on two sample functions: LeadingOnes and BinVal, are provided. The authors showed that there is also a phase transition from exponential to polynomial runtimes for ρ∼1/(nlogn) using the two sample functions. Furthermore, in [36], Gutjahr gave a more specific result and proof that with probability $2^{-\Omega (min(1/n\rho ,n))}$ the optimization time of 1-ant on LeadingOnes is $2^{\Omega (min(1/n\rho ,n))}$. Later, Zhou [37] studied the influence of pheromone control parameter α and heuristic information control parameter β on the computation time of the algorithm by constructing the traveling sales person problem (TSP) instances, which is a well-known NP-hard problem. It is showed clearly that for a complete graph instance, when the parameter is set from α = 1, β = 0 to α = 1, β = 1, the upper bound of its expected computing time is also changed from $O(n^{6})$ to $O(n^{5})$. In addition, Kötzing et al. [38] analysed the influence of heuristic information on the performance of the algorithm by constructing the TSP instance. It is stated that with α =1, If β = 1, the algorithm needs exponential run time to find the optimal tour, while with β=n, it finds the optimum in one iteration with almost the probability of 1.

Apparently the ant colony algorithm has less parameters to be pre-defined, but the optimal selection of these parameters is complicated. The current theoretical analysis is mainly focusing on for 1-ANT ant colony system assuming one ant in artificial pseudo-Boolean functions. Thus it turns to be a classical combinatorial optimization problem. For example, it is hard to analyze the influence of different parameter settings on algorithm performance. The optimization and theoretical analysis are still challenging problems when considering the multi-ant colony system.

In this paper, we propose a new strategy of using the minimal hop count to guide the path searching direction. We expect that it can greatly improve the convergence speed of the algorithm and reduce the computation time of finding the optimal solution. The procedure are given as follows.

We assume that the ant is currently on the node *i*. There are *n* nodes in $Set_{all}$. Firstly the following terms are defined:

$Set_{cur}$ is the set of the nodes whose hop counts are same as that of the node *i*;
$Set_{far}$ is the set of nodes whose hop counts are one more than that of the node *i*;
$Set_{near}$ is the set of the nodes whose hop counts are one less than that of the node *i*;
$Set_{all}$ is the set of all alternative next hop nodes.

According to the assumption, when the traditional ACO selects the next hop node for this ant, it calculates the node state transition probability for the *n* nodes in $Set_{all}$ respectively by using the probabilistic node state transition formula. Differently, our ACOHCM firstly puts the *n* nodes from $Set_{all}$ into $Set_{cur}$, $Set_{far}$ or $Set_{near}$ respectively according to their hop counts. $Set_{all}=Set_{cur}⋃Set_{far}⋃Set_{near}$. We also Assume that there are $num_{c}$ nodes in $Set_{cur}$, $num_{f}$ nodes in $Set_{far}$ and $num_{n}$ nodes in $Set_{near}$. Then $num_{c}+num_{f}+num_{n}=n$, $num_{c}$, $num_{f}$, and $num_{n}$ must be larger than 0. Then it can fall into the following two cases.

**Case** **1.** *When all n nodes are in the same set of* $Set_{cur}$, $Set_{far}$ or $Set_{near}$, *it selects the next hop in n nodes and calculates the state transition probability for the n nodes. The convergence speed of the algorithm does not improve*.

**Case** **2.** *When all n nodes are not in the same set, then at most one set is empty*. $num_{c}$, $num_{f}$, *and* $num_{n}$ *are all less than n. There are three following situations*:

*i*  *If* $num_{n}$ *> 0 and the energy of the nodes in* $Set_{near}$ *meets the requirements of data transmission, the algorithm only chooses the next hop nodes in* $Set_{near}$ *and calculates the node state transition probability for the nodes in* $Set_{near}$. *Therefore, the convergence speed of the algorithm can be improved by* $n\prime num_{n}$ *times*.
*ii*  *If* $Set_{near}$ *is empty or the energy of the nodes in* $Set_{near}$ *does not meet the requirements of data transmission, but* $num_{c}$ *> 0 and the energy of the nodes in* $Set_{cur}$ *meets requirements of data transmission, then the algorithm only chooses the next hop nodes in* $Set_{cur}$ *and only calculates the node state transition probability for the nodes in* $Set_{cur}$. *Therefore, the convergence speed of the algorithm can be improved by* $n\prime num_{c}$ *times*.
*iii*  *If* $Set_{near}$ *is empty and the energy of the nodes in* $Set_{cur}$ *does not meet the requirements of data transmission, or* $Set_{cur}$ *is empty and* $Set_{near}$ *is not empty but the energy of the nodes in* $Set_{near}$ *does not meet the requirements of data transmission, it is clear that* $num_{f}$ *> 0. If the energy of the nodes in* $Set_{far}$ *meets the requirements of data transmission, then the algorithm only chooses the next hop nodes in* $Set_{far}$ *and only calculates the node state transition probability for the nodes in. Therefore, the convergence speed of the algorithm can be improved by* $n\prime num_{f}$ *times*.

Since $num_{c}$, $num_{f}$, and $num_{n}$ are all far less than *n*, the convergence speed of ACOMHC algorithm can be greatly improved. In addition, using the idea of minimal hop count to guide the path searching direction of the next hop node selection can greatly reduce the total computational time on calculating the node state transition probability, which is also conducive to the improvement of the convergence speed. Moreover, the performance of the algorithm can be improved significantly by optimizing the parameters α,β,ρ and the heuristic function. Our theoretical analysis coincides the experimental results that present an impressive performance of the ACOMHC algorithm.

## 5. Experimental Results and Analysis

### 5.1. Simulation Settings of the WSNs

In order to evaluate the performances of ACOHCM algorithm, a simulation of WSN environment is run in C++. In our simulated network, the area of 100 m × 100 m, 100 nodes are randomly generated. As shown in Figure 1, the sink node is assumed at the center of the whole area. The best combination of the parameters α,β,ρ,Q in the algorithm is determined by experiments. These parameters in our simulation are shown in Table 1.

### 5.2. Comparison of Convergence Characteristics of the Several Algorithms

In the WSNs, due to the limited energy power of nodes and the dynamic network topology, it is critical that the routing protocol to calculate the optimal path with low cost. Therefore, the routing algorithms should have a high convergence speed and a high success rate of searching for the optimal solution.

**Figure 2:**
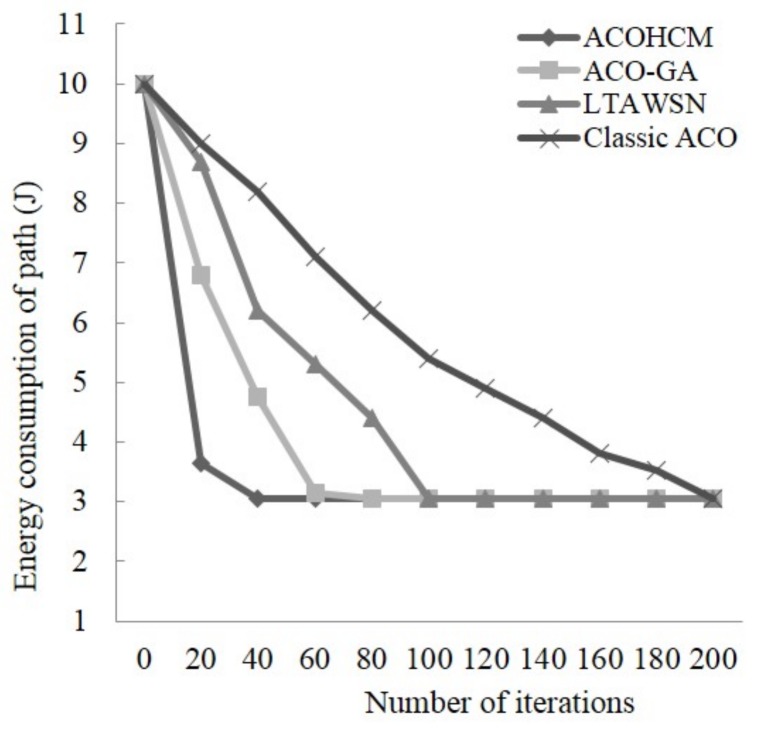
Convergent features of the different algorithms.

We conduct experiments that compare the convergence speed of the several algorithms including the classic ACO, LTAWSN, ACO-GA and ACOHCM. These four algorithms are run 100 times separately and Figure 2 reports the average number of iterations of each algorithm. In Figure 2, it is clearly to show that ACOHCM algorithm finds the optimal solution after 38 iterations on average, while classic ACO needs 198 iterations on average, 102 for LTAWSN, and 64 for ACO-GA. Therefore, it proves that ACOHCM algorithm is superior to the other three algorithms in terms of the convergence speed.

The improvement of the convergence speed of the ACOMHC algorithm is because of the following two reasons: on one hand, it owes to the strategy of using the minimal hop count to guide the path searching direction of the next hop node selection in the hop count-classification topology. On another hand, it owes to the role of pheromone, that is, to increase the pheromone on the optimized path in a certain range, we can speed up the convergence.

The average time for one iteration of the four algorithms and the success rate of searching for the optimal solution in 100 iterations of the four algorithms are recorded in Table 2. Table 2 shows that ACOHCM algorithm with hop count-classification topology is apparently superior to the other three in computing time. In order to test the success rate of searching for the optimal solution of the algorithms, each algorithm is tested 100 times respectively with 100 iterations in each test. As Table 2 shows that ACOHCM has a strong ability to search for the global optimal solution, and its success rate is higher than that of the other algorithms.

The above simulation results have shown that ACOHCM can greatly improve the efficiency of routing computation and the success rate of searching for the global optimal solution, and verify that ACOHCM algorithm is reliable and effective.

### 5.3. Comparison of the Network Lifetime

As we mentioned before, the network lifetime is one of the most important indicators of WSNs. The energy of a node in a WSN is mostly spent on data transmission. Especially, the nodes who are responsible for data forwarding in their region. If these nodes are out of power, data will not be able to be normally forwarded to the sink node. Therefore, energy exhausting of a node in WSNs may affect the normal operation of the whole network. In this paper, the network lifetime is defined as the duration beginning when the network is activated until when one node’s battery goes flat.

**Figure 3:**
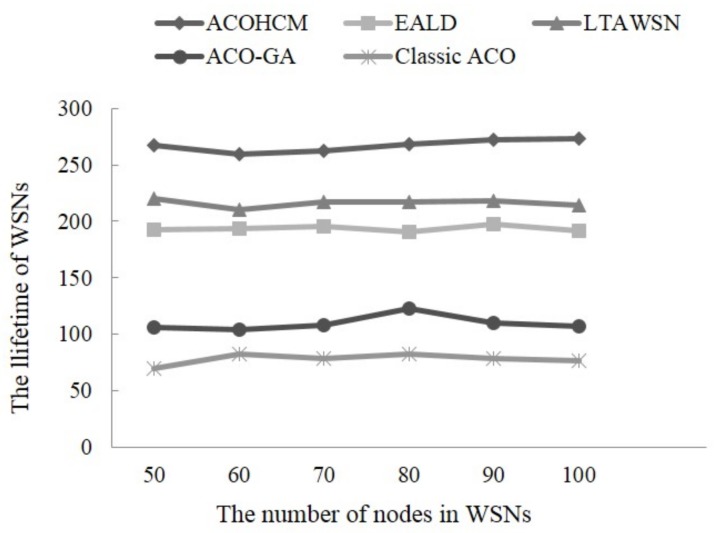
The network lifetime of the different algorithms changing with the number of nodes.

Therefore we simulate and compare the network lifetime of the classic ACO, LTAWSN, ACO-GA, EALD, and ACOHCM algorithm in the experiments as follows. The networks which consist of 50, 60, 70, 80, 90, and 100 sensor nodes are generated respectively in the simulation area, and The nodes’ routing information is produced by running five algorithms respectively. Figure 3 is the comparison of the network lifetime of our proposed one against other four algorithms. It is clearly to see that the network lifetime of ACOHCM is the longest. The main reason why ACOHCM algorithm can extend the network lifetime at a maximum extent is that it uses the dynamic energy threshold strategy to take a strict control of the node energy consumption, trying to avoid the low energy nodes in the process of path selection. On another hand, the proposed algorithm finds the best path with minimum total energy consumption in the energy-abundant nodes.

## 6. Conclusions

This paper proposes an effective hybrid WSN routing algorithm named ACOHCM. Different from the existing WSN routing algorithms that only consider one aspect of improvement in terms of minimizing total power consumption, balancing the network load, speeding up the convergence and the dynamic networking capability. Our proposed ACOHCM takes into comprehensive account energy efficiency, load balancing, convergence speed, and routing reliability as well as ease of maintenance so as to fulfill its use in real-world applications. The algorithm combines ant colony optimization with minimum hop count routing strategy, greatly improving the efficiency and overall performance. In order to search for the optimal routing path, in consideration of the characteristics of WSNs, we revise the heuristic factor and replace a new pheromone update strategy in the probabilistic node state transition formula for path selection in the ant colony algorithm. In addition, we propose a dynamic energy threshold strategy and a mutation strategy. The proposed algorithm is found to be efficient and it effectively prolongs the network lifetime in comparison with other methods such as classic ACO, LTAWSN, ACO-GA and EALD.

References

## Figures and Tables

**Table 1:** Parameters in the simulation.

Parameter	Value	Parameter	Value
$E_{elec}$	50 nJ/bit	$\epsilon_{mp}$	0.0013 (pJ/bit/m${}^{4}$)
$\epsilon_{fs}$	10 (pJ/bit/m${}^{2}$)	$d_{max}$	20 m
*k*	100 KB	γ	2.5
*C*	1000 J	*Q*	100
α	0.5	β	5
ρ	0.3	$iter_{cnt}$	100
threshold	800 J	$threshold_{min}$	300 J
$\tau_{max}$	200	$\tau_{min}$	100

**Table 2:** Time consumption for iteration and the success rate of searching for the optimal solution of the algorithms.

Name of the Algorithms	Time per an Iteration	Success Rate
ACOHCM	3.46 ms	99%
ACO-GA	5.79 ms	92%
LTAWSN	8.71 ms	88%
Classic ACO	15.63 ms	55%
